# Resources and Workforce in Top-Tier Psychiatric Hospitals in China: A Nationwide Survey

**DOI:** 10.3389/fpsyt.2021.573333

**Published:** 2021-02-24

**Authors:** Lei Xia, Feng Jiang, Jeffrey Rakofsky, Yulong Zhang, Yudong Shi, Kai Zhang, Tingfang Liu, Yuanli Liu, Huanzhong Liu, Yi-lang Tang

**Affiliations:** ^1^Department of Psychiatry, Chaohu Hospital of Anhui Medical University, Hefei, China; ^2^Department of Psychiatry, Anhui Psychiatric Center, Anhui Medical University, Hefei, China; ^3^Healthcare Management and Evaluation Research Center, Institute of Health Yangtze River Delta, Shanghai Jiao Tong University, Shanghai, China; ^4^Research Center for Public Health, Tsinghua University, Beijing, China; ^5^Department of Psychiatry and Behavioral Sciences, Emory University, Atlanta, GA, United States; ^6^Institute for Hospital Management of Tsinghua University, Beijing, China; ^7^Public Health School, Chinese Academy of Medical Sciences and Peking Union Medical College, Beijing, China; ^8^Atlanta Veterans Affairs Medical Center, Decatur, GA, United States

**Keywords:** mental health, resources, workforce, psychiatric hospitals, China

## Abstract

**Objectives:** Mental healthcare has gained momentum and significant attention in China over the past three decades. However, many challenges still exist. This survey aimed to investigate mental health resources and the psychiatric workforce in representative top-tier psychiatric hospitals in China.

**Methods:** A total of 41 top-tier psychiatric hospitals from 29 provinces participated, providing data about numbers and types of psychiatric beds, numbers of mental health professionals, outpatient services and hospitalization information covering the past 3 years, as well as teaching and training program affiliation.

**Results:** Significant variations were found among participating hospitals and across different regions. Most of these hospitals were large, with a median number of psychiatric beds of 660 (range, 169-2,141). Child and geriatric beds accounted for 3.3 and 12.6% of all beds, respectively, and many hospitals had no specialized child or geriatric units. The overall ratios of psychiatrists, psychiatric nurses, and psychologists per bed were 0.16, 0.34, and 0.03, respectively. More than 40% of the hospitals had no clinical social workers. Based on the government's staffing guidelines, less than one third (31.7%) of the hospitals reached the lower limit of the psychiatric staff per bed ratio, and 43.9% of them reached the lower limit of the nurse per bed ratio.

**Conclusion:** Although some progress has been made, mental health resources and the psychiatric workforce in China are still relatively insufficient with uneven geographical distribution and an acute shortage of psychiatric beds for children and elderly patients. In the meantime, the staffing composition needs to be optimized and more psychologists and social workers are needed. While addressing these shortages of mental health resources and the workforce is important, diversifying the psychiatric workforce, promoting community mental health care, and decentralizing mental health services may be equally important.

## Introduction

Psychiatry and mental health services in China have been traditionally marginalized with limited resources allocated (1–3), however, some positive progress has been noticed. In recent decades these areas have gained certain momentum and significant attention from the public and the government (4), in large part due to recent studies showing the high rates of mental disorders (5, 6) in the population, the increasing global burden of mental disorders, as measured by the disability adjusted life years (DALYs) (7), and the impact of mental disorders on labor market and workplace productivity (8). A recent national mental health survey (9) was conducted in 31 provinces across China and it found the lifetime prevalence of any mental disorder was 16.6%, while the 12-month prevalence was 9.3%. On the other hand, the estimated disability adjusted life years (DALYs) in thousands by mental and substance use disorders reached 31,095.0 years in China in 2016 (30,368.7 years in 2010, and 31,014.0 years in 2015), which accounted for 8.3% of the total disease burden of China, and 18.2% of the global burden of mental and substance use disorders (10).

Facing increasing challenges in mental health care, great efforts have been made in China to reform the mental health service system (4, 11). One milestone is China's very first Mental Health Law (MHL) (implemented in 2013) (12), which is comprehensive, and involves many aspects of mental health services, including the budget, resources, community services, patients' rights, and professional training. Subsequently, the National Mental Health Work Plan (2015–2020) (13) requires improving the system and network of mental health services and mandating each county (city, district) set up a psychiatric department in at least one general hospital. To increase the supply of the psychiatric workforce, 31 medical colleges across China have established psychiatry programs for medical students with vigorous promotion by the National Health Commission and Ministry of Education. These are specialized programs with a curriculum focusing on psychiatry-related teaching, with the goal to fast-track graduates to psychiatry residency directly (14). Now, there are total 6,271 medical students in psychiatry programs in medical colleges, and 720 students graduated in 2018.

With all these efforts, significant progress has been made in bolstering mental health resources and the psychiatric workforce across the country. The number of psychiatric beds nationwide increased from 228,100 (17.1 per 100,000 population, same units hereafter unless otherwise specified) in 2010 to 433,090 (31.5) in 2015, with a growth rate of 89.9% (15). There were 13 provinces with an increase of more than 100% in the number of psychiatric beds (Top three provinces: Guizhou with an increase of 241.4%, Jiangxi with 192.5%, and Hunan with 172.9%, respectively). The government has put forward a plan to strengthen the mental health service system in the central and western regions and on a national level (15). The numbers of psychiatrists and psychiatric nurses increased from 20,480 (1.54) and 35,337 (2.65) in 2010 (16) to 30,122 (2.19) and 75,765 (5.51) in 2015 (15), respectively. By the end of 2018, there were 506,637 psychiatric beds (36.3) and about 36,000 psychiatrists (2.58), including licensed psychiatrists and psychiatric registrars in China (17), which are slightly above the average (or median) levels in upper middle-income countries (UMICs) (24.3 and 2.11, respectively) (18).

However, several insufficiencies in mental health resources and workforce of China remain. First, for the most part, mental health services in China are organized around psychiatric hospitals, which provide nearly 80% of mental health services (78.8% of all beds, and 78.4% of mental health workers) in China, followed by psychiatric departments in general hospitals (16.8 and 17.3%), primary care settings (3.8 and 3.1%), and rehabilitation hospitals (1.4 and 0.9%) (15). This is very different from many other countries. The reality is, except for a few major cities such as Beijing, Shanghai and Guangzhou (Canton), community mental health services are rather limited and psychiatric hospitals remain as the main or even the only mental health provider in most places. Second, mental health resources are even more limited for key population subgroups, including the elderly and children. Third, relative to the mental health workforce located in UMICs, the total number of Chinese mental health workers is small (8.90 vs. 20.6 per 100,000 population) and staffing composition is different from that in UMICs (5.51 vs. 6.83 psychiatric nurses, 0.35 vs. 1.89 psychologists, and 0.11 vs. 0.50 social workers per 100,000 population) (15, 18), with psychologists and social workers disproportionally understaffed.

To date, no detailed data at the hospital level are available to enable suitable analysis and guidance on the allocation of mental health resources and the psychiatric workforce in China. Thus, the purpose of this study was to investigate and analyze the resources and the workforce based on several new indicators in representative top-tier psychiatric hospitals in China, and hope to use the data to optimize resource allocation and staffing to improve hospital-based mental health service in China.

## Methods

### Study Design and Participants

This study was part of the 2019 National Hospital Performance Evaluation Survey (NHPES). It focused on the topic of mental health services, aiming to identify current deficiencies and imbalances in China's mental health care and to ultimately improve its quality. Basic information about each hospital was filled out by staff members in the hospitals' medical management departments through an online questionnaire survey from March 18 to 31, 2019. A paper document with the hospital manager's signature, contact information, and hospital seal was subsequently requested to be submitted. As a result of the assurance of a notification issued by each Provincial Health Commission, participation rate of target hospitals reached 100%. The psychiatric investigation project had been approved by the Ethics Committee of Chaohu Hospital of Anhui Medical University (No. 201903-kyxm-02) before the study began.

**Figure 1:**
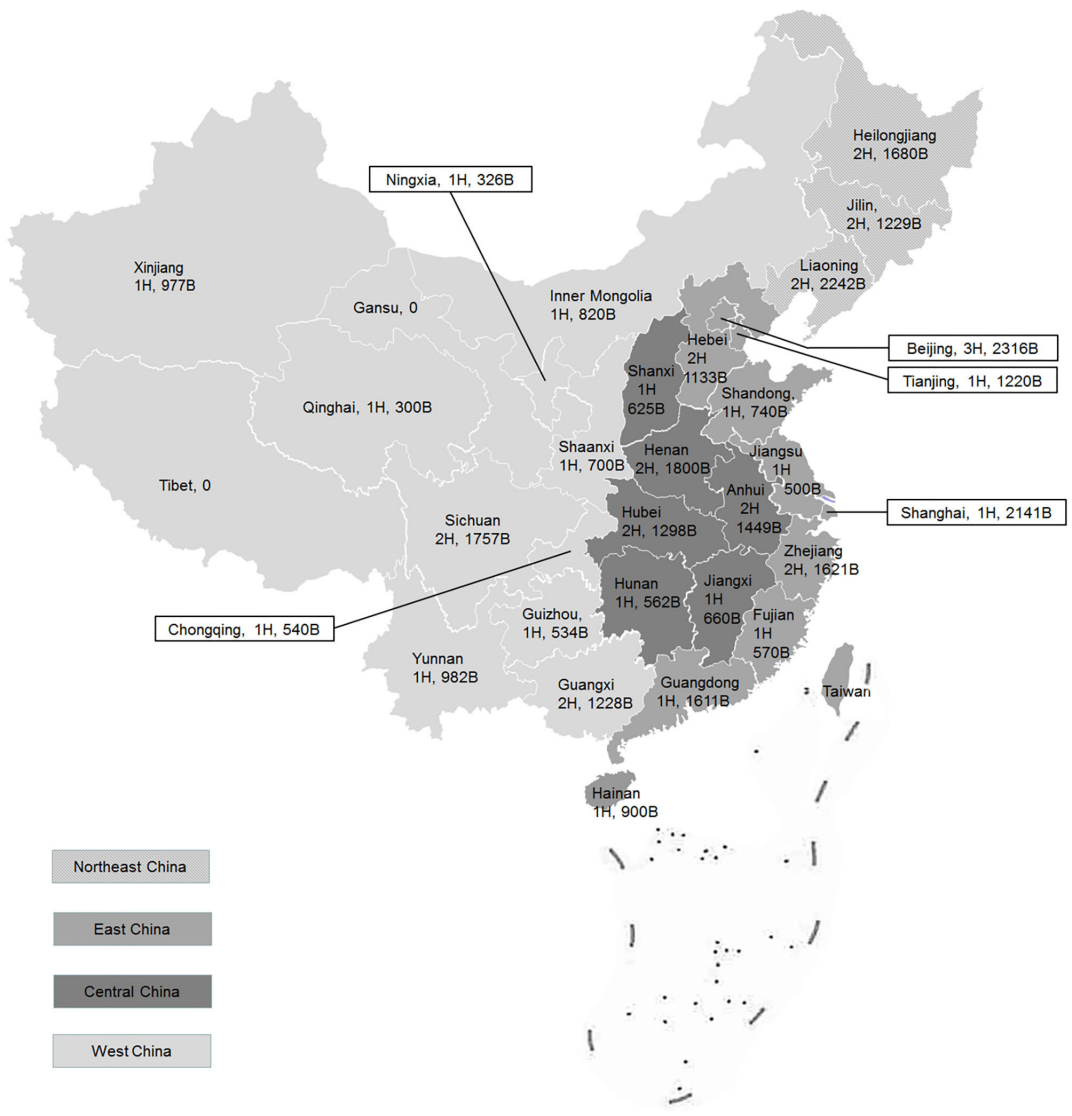
Geographical distribution and basic data of 41 participating psychiatric hospitals in China. H, number of hospitals; B, number of psychiatric beds.

In total, 41 tertiary psychiatric hospitals from 29 provinces and autonomous regions in China were investigated and coded as P1–P41. Two provinces, Gansu and Tibet, had no provincial tertiary psychiatric hospitals at the time of survey and therefore were not included. According to the classification of Chinese economic regions reported by the National Bureau of Statistics (19), four regions were ranked by the number of top-tier psychiatric hospitals: East China (14 hospitals), West China (12 hospitals), Central China (nine hospitals), and Northeast China (six hospitals) (Figure 1).

### Data Collection

The questionnaire about resources and the workforce was designed for these hospitals above to collect psychiatric bed data (i.e., number of psychiatric beds in closed-door, open-door, child, and geriatric wards, and occupancy rate), psychiatric workforce composition (i.e., number of psychiatrists, psychiatric nurses, psychologists, and social workers), outpatient services and hospitalization information (i.e., outpatient visits, average hospitalization days, and number of discharged patients) in the past 2 years, teaching and training program affiliation, and other relevant information. The supplementary hospitalization information of 32 hospitals (P1–P32) in 2016 was extracted from the NHPES conducted in 2017 (20).

To exclude resources related to non-psychiatric services provided in 15 of the 41 hospitals (P12, P16, P21, P22, P23, P24, P26, P27, P32, P34, P35, P36, P37, P38, and P41), we conducted a second survey from October 18 to 31, 2019. Data about psychiatric standardized residency training programs (2019) were obtained from their official websites in each province. All data collection and entry were double checked.

### Indicators About Staffing and Setting

We collected the numbers of psychiatric beds (including beds in closed-door wards and open-door wards), psychiatrists, psychiatric nurses, psychologists/ therapists and social workers in each hospital. Based on these numbers, we calculated different ratios as indicators of staffing adequacy and composition: They include: the number of psychiatrists/number of beds (P/B ratio); psychiatric nurses/beds (N/B ratio); psychiatric nurses/psychiatrists (N/P ratio). The staffing guidelines in tertiary psychiatric hospitals (21) recommended by National Health Commission of China was as follows: at least 0.55 healthcare professionals per bed with an average of at least 0.35 nurses per bed. Because most of the time, doctors and nurses are the primary providers working directly with patient care in the inpatient setting in China, the sum of psychiatrists and psychiatric nurses was substituted for the number of healthcare professionals in the data analysis. Variation in outpatient visits, average hospitalization days, and number of discharged patients in these psychiatric hospitals from 2016 to 2018 were also calculated as they reflect the current need for mental health services in China. In addition, the psychiatrists in these hospitals were required to provide mental health services in the outpatient clinic for outpatients, therefore, the ratio of outpatient visits (2018 data) /psychiatrists (OVs/P) was calculated for measuring the overall productivity of the psychiatrists' outpatient work.

### Data Analysis

Statistical descriptions and analyses were conducted using IBM SPSS, version 23.0. Frequency distributions and medians were calculated for qualitative and quantitative variables, respectively, and weighted ratios for P/B, N/B, N/P, and OVs/P. Shapiro-Wilk test was used to detect the distribution normality of variables (*n* < 50). Outpatient visits (OVs), average hospitalization days (AHDs), and number of discharged patients (DPs) between adjacent years were compared by using Paired-Samples *T* Test or Two Related-Samples Test (Wilcoxon), as appropriate. The statistical significance was set at *p* < 0.05 (2- tailed).

## Results

### Psychiatric Bed Settings

The median number of total psychiatric beds was 660 (range, 169-2,141) in 41 top-tier psychiatric hospitals in China (Table 1). 80.5% (33/41) had ≥500 psychiatric beds, nearly two thirds (26/41, 63.4%) had a bed occupancy rate of over 100 percent. The median number of psychiatric beds in closed-door wards and open-door wards were 535 (range, 124–2093) and 120 (range, 20–452), respectively. Regarding the psychiatric bed settings for special populations, we found that 75.6% (31/41) of these hospitals had a child psychiatric unit and 90.2% (37/41) had a geriatric unit. Overall, child and geriatric beds only accounted for 3.3 and 12.6% of all psychiatric beds, respectively.

**Table 1:** Number of psychiatric beds and staff composition in 41 top-tier psychiatric hospitals in China.

	**Number of psychiatric beds**						**Psychiatric staff**							
**Hospital code**	**Location**	**Total** **beds (*n*)**	**Occupancy** **rate (*%*)**	**Closed-door** **beds (n)**	**Open-door** **beds (n)**	**Child** **beds (*n*)**	**Geriatric** **beds (*n*)**	**Psychiatrists** **(*n*)**	**P/B**	**Nurses** **(*n*)**	**N/B**	**N/P**	**Psycho-** **logists** **(*n*)**	**Social** **Worker** **(*n*)**
P01	Beijing	221	105.18	160	61	30	29	80	0.36	127	0.57	1.59	0	4
P02	Beijing	800	111.83	780	20	57	51	245	0.31	386	0.48	1.58	9	8
P03	Beijing	1,295	111.84	1,203	92	12	199	176	0.14	530	0.41	3.01	34	6
P04	Tianjin	1,220	102.60	1,124	96	32	102	211	0.17	467	0.38	2.21	12	8
P05	Hebei	523	116.30	378	145	0	0	89	0.17	173	0.33	1.94	4	0
P06	Shanxi	625	80.91	553	72	10	72	97	0.16	230	0.37	2.37	10	0
P07	Inner Mongolia	820	101.87	631	189	35	60	222	0.27	421	0.51	1.90	11	1
P08	Liaoning	1,532	143.80	1,466	66	58	66	159	0.10	385	0.25	2.42	8	23
P09	Jilin	800	74.40	556	244	0	56	100	0.13	285	0.36	2.85	26	3
P10	Heilongjiang	1,250	77.04	798	452	0	0	132	0.11	333	0.27	2.52	4	1
P11	Shanghai	2,141	113.37	2,093	48	25	314	231	0.11	584	0.27	2.53	10	4
P12	Jiangsu	500	106.00	420	80	30	80	135	0.27	231	0.46	1.71	91	0
P13	Zhejiang	1,054	99.43	845	209	32	350	194	0.18	338	0.32	1.74	13	0
P14	Anhui	169	128.30	124	45	10	10	28	0.17	34	0.20	1.21	17	14
P15	Anhui	1,280	116.85	1,005	275	74	77	177	0.14	390	0.30	2.20	145	1
P16	Fujian	570	97.58	474	96	20	90	65	0.11	152	0.27	2.34	14	1
P17	Jiangxi	660	103.80	594	66	0	40	134	0.20	268	0.41	2.00	35	0
P18	Shandong	740	104.70	530	210	35	35	153	0.21	289	0.39	1.89	20	0
P19	Henan	468	125.40	350	118	45	60	155	0.33	203	0.43	1.31	47	11
P20	Hubei	950	115.00	930	20	65	65	174	0.18	360	0.38	2.07	24	1
P21	Hunan	562	95.10	447	115	26	108	84	0.15	169	0.30	2.01	15	28
P22	Guangdong	1,611	98.12	1,491	120	40	210	226	0.14	494	0.31	2.19	13	7
P23	Guangxi	590	109.60	460	130	10	40	128	0.22	206	0.35	1.61	5	0
P24	Hainan	900	85.53	800	100	0	42	116	0.13	281	0.31	2.42	20	0
P25	Chongqing	540	117.91	495	45	0	150	117	0.22	245	0.45	2.09	30	0
P26	Sichuan	1,257	97.00	1,134	123	12	350	173	0.14	313	0.25	1.81	176	0
P27	Guizhou	534	81.15	380	155	5	120	74	0.14	174	0.33	2.35	6	0
P28	Yunnan	982	102.80	773	209	40	380	140	0.14	282	0.29	2.01	5	0
P29	Shaanxi	700	106.14	665	35	45	65	120	0.17	148	0.21	1.23	8	0
P30	Qinghai	300	74.50	185	109	0	0	65	0.22	99	0.33	1.52	7	1
P31	Ningxia	326	87.37	300	26	0	0	71	0.22	163	0.50	2.30	32	3
P32	Xinjiang	977	129.54	535	442	35	245	108	0.11	296	0.30	2.74	14	0
P33	Hebei	610	109.22	369	241	15	82	128	0.21	252	0.41	1.97	33	9
P34	Liaoning	710	87.20	540	170	0	80	49	0.07	164	0.23	3.35	21	0
P35	Jilin	429	92.60	150	279	0	46	54	0.13	136	0.32	2.52	7	1
P36	Heilongjiang	430	84.00	400	30	5	50	38	0.09	143	0.33	3.76	2	0
P37	Henan	1,332	100.55	1,103	229	73	55	199	0.15	498	0.37	2.50	45	0
P38	Sichuan	500	101.10	370	130	120	74	69	0.14	129	0.26	1.87	16	1
P39	Zhejiang	567	110.41	200	367	6	188	89	0.16	199	0.35	2.24	3	0
P40	Hubei	348	139.50	147	201	30	30	35	0.10	83	0.24	2.37	2	1
P41	Guangxi	638	103.30	478	160	35	35	84	0.13	261	0.41	3.11	11	2
Total		32,461	-	26,436	6,020	1,067	4,106	5,124	0.16	10,921	0.34	2.13	1,005	139

P/B, number of psychiatrists per bed; P/B, number of psychiatric nurses per bed.

*N/P, number of psychiatric nurses/number of psychiatrists*.

### Psychiatric Workforce and Its Composition

The median number of psychiatrists and psychiatric nurses were 117 (range, 28–245) and 245 (range, 34–584) in these hospitals (Table 1). The overall ratios of P/B and N/B were 0.16 and 0.34 per bed, respectively, with wide variations across different hospitals (from 0.07 to 0.36, and 0.20 to 0.57). Using the staffing guidelines set by the government, less than one third (31.7%) of the hospitals reached the lower limit of the staffing ratio, and 43.9% of them reached the lower limit of nurses per bed. The overall ratio of N/P was around 2:1, and again with a wide variation among different hospitals (from 1.21 to 3.76, more than three times).

**Figure 2:**
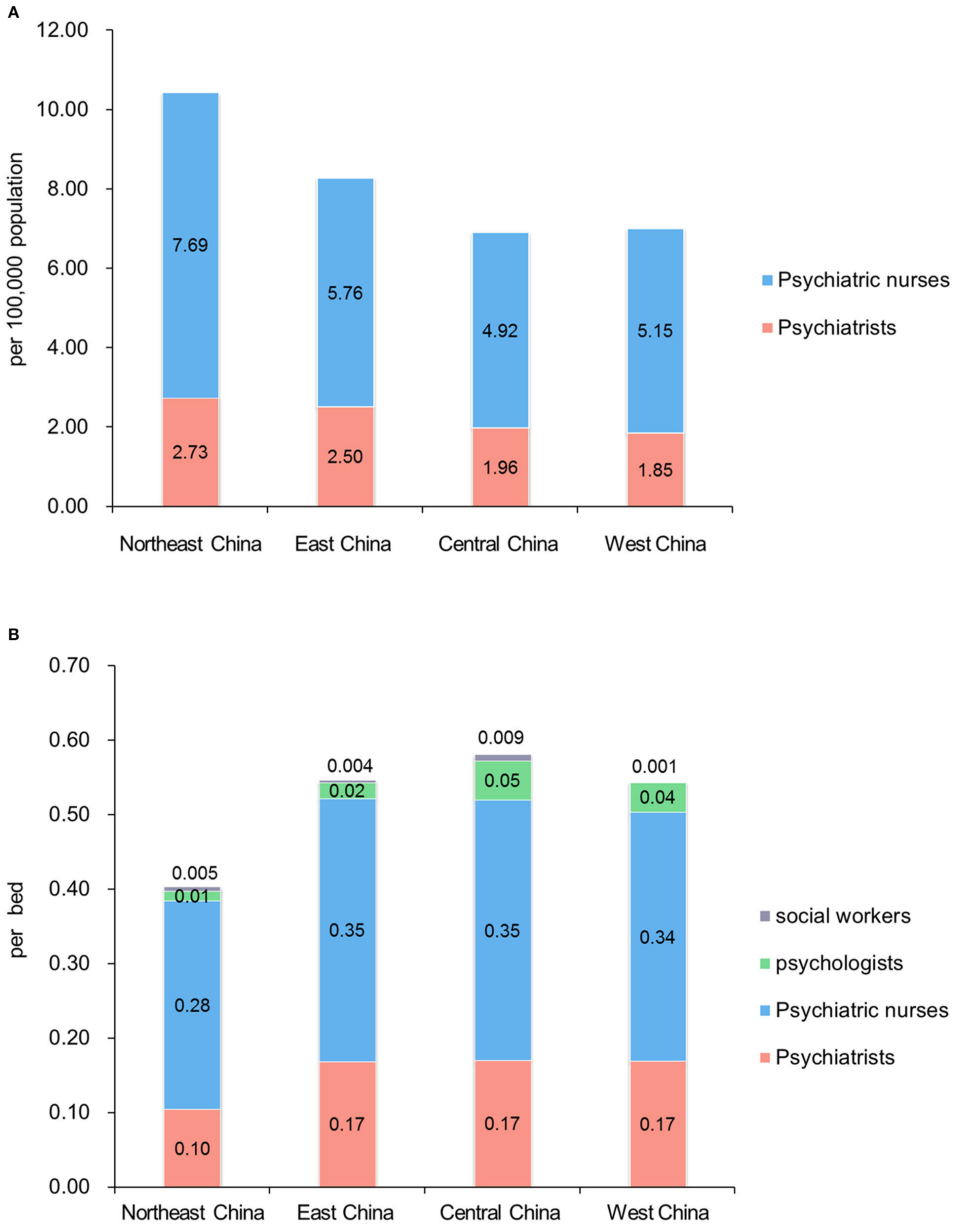
Comparison of mental health workforce in different regions in China, and data from this survey. **(A)** Mental health workforce (psychiatrists and nurses only) in different regions in China (per 100,000 population). Staffing data from the national survey at end of 2015, and provincial demographic data from Chinese Health Statistical Yearbook 2019. Numbers of other mental health workers by region were not available. **(B)** Psychiatric workforce in different regions in 41 top-tier psychiatric hospitals (per bed).

The total number of psychologists was 1,005, and the ratio of psychologists to beds was 0.03 per bed. Notably, 43.9% (18/41) of these hospitals had no social workers. In all, the number of social workers was only 139 in these 41 top-tier psychiatric hospitals. Table 2 showed the staffing composition in UMICs, China, and this survey, and Figure 2 showed the comparison of mental health workforce in different regions in China and this survey.

**Table 2:** Staffing composition in upper middle income countries (UMICs), China, and data from this survey.

	**UMICs** **(per 100,000 population)**^**a**^	**China(per 100,000 population)^**b**^**	**Top-tier psychiatric** **hospitals (per bed)^**c**^**
Psychiatrists	2.11	2.19	0.16
Psychologists	1.89	0.35	0.03
Nurses	6.83	5.51	0.34
Social workers	0.50	0.11	0.004
Occupational therapists	0.24	0.08	Not applicable
Other workers	2.29	0.66	Not applicable

a Mental health workforce in UMICs (per 100,000 population). Data from Mental health Atlas 2017.

b Mental health workforce in China (per 100,000 population). Staffing data from the national survey of mental health resource at end of 2015 (Published in 2019).

c *Psychiatric workforce in 41 top-tier psychiatric hospitals (per bed)*.

### Outpatient Services and Hospitalization

Overall, OVs, and DPs increased from 2016 to 2018, while AHDs decreased (Table 3). The medians of ΔOVs, ΔDPs, and ΔAHDs were 32,836 (range, −18,718–117,110), 924 (range, −1,455–7,582), and −3.04 (range, −51.85–72.10) in 32 hospitals, respectively. Significant statistical differences were observed in OVs (*t* = −2.931, *p* = 0.006), DPs (*Z* = −4.226, *p* < 0.001), and AHDs (*Z* = −2.001, *p* = 0.04*5*) between 2016 and 2017, and OVs (*t* = −6.242, *p* < 0.001), DPs (*Z* = −4.231, *p* < 0.001), and AHDs (*Z* = −3.217, *p* = 0.001) between 2017 and 2018, which reflects the demands for psychiatric outpatient services and hospitalization increasing year by year. After removal of the hospitals that had more than three other non-psychiatric outpatient or clinical departments, the differences remained significant in OVs (*t* = −2.978, *p* = 0.006), and DPs (*Z* = −3.619, *p* < 0.001) between 2016 and 2017, and OVs (*t* = −5.604, *p* < 0.001), DPs (*t* = −3.798, *p* < 0.001), and AHDs (Z = −2.303, *p* = 0.021) between 2017 and 2018.

**Table 3:** Outpatient visits, average hospitalization days, and discharged patients in 41 top-tier psychiatric hospitals in China from 2016 to 2018.

	**Outpatient visits** **(2016–2018)**			**Average hospitalization days** **(2016–2018)**			**Discharged patients** **(2016–2018)**		
**Hospital code**	**OVs-2016 (*n/year*)**	**OVs-2017(*n/year*)**	**OVs-2018 (*n/year*)**	**AHDs-2016(*days*)**	**AHDs-2017 (*days*)**	**AHDs-2018(*days*)**	**DPs-2016 (*n/year*)**	**DPs-2017(*n/year*)**	**DPs-2018 (*n/year*)**
P01	292,810	302,199	324,163	28.01	26.41	25.55	3,027	3,238	3,311
P02	452,875	495,353	553,007	34.28	37.32	38.08	7,399	7,964	8,303
P03	135,124	153,831	173,083	87.21	75.72	69.20	5,831	6,824	7,634
P04	458,830	519,312	566,366	68.80	67.80	67.50	7,147	6,934	6,786
P05	47,184	54,677	64,926	40.70	39.70	36.60	3,822	4,460	5,864
P06	31,824	53,948	94,114	80.50	78.31	63.66	2,577	2,804	2,854
P07	176,807^b^	176,833	200,848	63.63	51.80	50.00	3,586	5,122	5,900
P08	159,855	184,305	215,928	135.00	140.00	124.00	5,345	5,305	5,070
P09	122,989	150,947	181,201	27.20	26.00	26.60	7,351	7,669	7,765
P10	151,067	163,946	185,386	25.55	22.57	20.52	15,978	17,818	17,306
P11	807,900	857,429	922,673	68.00	107.69	140.10	6,514	6,694	7,041
P12^a^	788,940	854,785	906,050	19.00	18.70	18.70	20,747	21,378	22,194
P13	314,356	354,521	399,595	21.57	20.91	20.33	15,920	17,993	18,776
P14	18,470	26,007	34,748	56.70	50.90	45.30	859	1,362	1,802
P15	218,337	233,061	273,457	46.20	55.98	61.55	8,372	9,276	8,389
P16^a^	315,583	277,218	296,865	37.15	35.38	35.05	6,354	6,443	6,408
P17	127,356	122,703	110,902	60.60	66.10	62.50	3,754	3,783	3,742
P18	175,423	204,305	249,127	62.00	57.41	46.80	4,172	4,768	6,031
P19	160,901	171,569	202,338	50.52	55.91	46.91	4,096	4,195	4,462
P20	235,723	309,540	343,259	50.16	43.22	32.76	9,102	9,751	11,653
P21^a^	295,545	287,104	317,928	15.90	14.60	13.60	33,472	36,656	41,054
P22	599,371	539,344	644,919	82.36	33.17	30.51	6,907	7,128	8,940
P23^a^	85,232	94,794	110,944	44.80	38.10	36.80	5,579	5,946	6,448
P24	169,348	141,819	157,640	58.00	48.89	44.02	3,798	5,136	5,654
P25	96,179	79,942	93,870	67.28	66.95	59.39	5,389	3,567	3,934
P26^a^	188,556	251,013	300,121	56.90	54.37	51.25	7,984	9,731	10,190
P27^a^	189,952	180,054	188,187	19.36	18.55	17.01	9,454	11,587	13,570
P28	246,083	258,217	247,812	47.90	44.50	46.80	6,466	7,572	7,970
P29	94,059	108,374	117,561	36.90	36.34	36.30	5,834	5,892	6,441
P30	43,691	52,242	61,910	58.70	35.99	44.53	1,097	1,358	1,947
P31	42,221	54,580	61,317	48.93	55.25	47.58	1,672	2,117	2,173
P32	208,376	224,060	200,652	32.00	31.00	36.00	10,099	11,627	11,349
P33	-	118,540	127,908	-	35.34	35.70	-	6,951	6,877
P34^a^	-	40,855	44,144	-	110.00	94.00	-	2,162	2,354
P35^a^	-	186,595	189,215	-	16.18	15.60	-	17,444	18,860
P36^a^	-	118,141	120,719	-	40.00	30.00	-	10,996	11,436
P37	-	209,636	229,470	-	45.04	40.53	-	12,134	12,856
P38^a^	-	599,210	679,751	-	15.10	14.80	-	47,124	49,229
P39	-	110,227	127,063	-	30.92	28.20	-	6,882	8,015
P40	-	131,621	162,201	-	16.60	16.60	-	6,165	6,896
P41^a^	-	200,253	186,237	-	26.30	24.00	-	14,768	14,589
Total	7,450,967	9,653,110	10,667,605	1,631.81	1,891.02	1,794.93	239,704	386,724	412,073

OVs, outpatient visits; AHDs, average hospitalization days; DPs, Discharged patients.

a These hospitals had more than three other non-psychiatric outpatient or clinical departments, data related to which were included.

b *Corrected datum*.

In addition, the ratio of OVs/P was 1,730.5, ranging from 729.5 to 4,634.3 in 26 psychiatric hospitals with only psychiatric outpatient departments. Assuming all listed psychiatrists worked at outpatient clinics for 104 days/year (2 days a week), then each psychiatrist saw between 7 and 45 patients every working day. Considering many psychiatrists do not work in the outpatient clinic, the actual caseload of each psychiatrist was much higher than this.

### Psychiatric Medical Education and Standardized Residency Training

Ninety point two percent (37/41) of the hospitals surveyed were teaching hospitals affiliated with a medical college. Eighty seven point eight percent (36/41) had a psychiatric residency training program, and there were 391 trainees (N/A in three hospitals) in 2019. In all, 1,322 psychiatric residents were newly recruited in the 26 provinces and regions (N/A in three provinces) of China in 2019 (Supplementary Table 1). There were large differences in the numbers of psychiatric residents in provincial recruitment plans with the most (749 psychiatric residents) recruited from provinces in East China, a socioeconomically developed region.

## Discussion

In this study, significant efforts were taken to collect accurate data about mental health resources and the psychiatric workforce in 41 top-tier psychiatric hospitals from 29 provinces and autonomous regions across China. All of these hospitals are located in urban centers, representing the best psychiatric resources available in China.

Our findings are both encouraging and concerning. While we found the overall number of mental health professionals in top-tier psychiatric hospitals has increased over the past decade, a few areas clearly need improvement and change. The results showed that although 80% of these hospitals had more than 500 beds, more than 60% still had a bed occupancy rate of over 100 percent, an indicator that more psychiatric beds are needed or more community resources need to be developed. When “extra or temporary” beds are added to accommodate more patients or for economic reasons, safety and security problems may arise.

The non-existence or shortage of child and geriatric psychiatric beds in some provinces or regions is very concerning as well. Due to their unique developmental features, children and adolescents who require inpatient care for mental health problems should be managed in age-appropriate facilities (22). Similarly, due to their special needs in mobility, cognition and other age-related issues, elderly patients often need to be in a specially designed and staffed unit.

According to the most recent data (23), by the end of 2018, the number of children (aged 0–14 years) in China reached 16.9% of the Chinese population (1.395 billion); and the number of elderly (aged ≥65 years) reached 11.9%, and it has been growing for the past 23 years. The prevalence of mental disorders are high in both children (9–10%) (24, 25) and the elderly (4.9%) (9). One recent report showed that there were only 3,825 child psychiatric beds (0.89%) and 28,118 geriatric psychiatric beds (6.49%) nationwide in China (2015 data) (15). In this study, we found 10/41 (24.4%) hospitals did not have a child unit, and 4/41 (9.8%) did not have either a child or a geriatric unit. The overall child and geriatric beds accounted for 3.3 and 12.6% of all beds, respectively. Although they were expectedly higher than the national rate (0.89 and 6.49%, respectively), they were still quite limited. It is known that many children with mental disorders are rejected from hospitals or they are hospitalized with adults, causing safety and other concerns. According to a recent report, there are fewer than 500 full-time child psychiatrists in China, and the distribution is uneven (26).

Of note, we found that the number of psychiatrists and psychiatric nurses relative to beds was small in top-tier psychiatric hospitals, although these numbers have increased over the past few years on a national scale. There was also a very limited number of psychologists in these hospitals (0.03 per bed, one psychologist for 30 beds), and there were virtually no psychologists in some hospitals, or psychologists working on inpatient units. According to the estimated number of mental health workers needed in 44 middle-income countries (MICs) (1.5 psychiatrist, 15.0 nurses, and 10.2 psychosocial care providers per 100,000 population) (15, 27), there was a serious shortage of 135,000 psychologists/therapists (9.85 per 100,000) in China by the end of 2015.

Mental health services involve multidisciplinary teams. In addition to psychiatrists and nurses, the roles of psychologists, social workers, and peer specialists are very important and even essential (28, 29). With effective short-term training and supervision, non-specialist health workers including social workers and lay workers may contribute to mental health services in psychiatric hospitals and other settings (29, 30). In this survey, 43.9% (18/41) of the hospitals did not have a single social worker, and only 9.8% (4/41) had more than 10 social workers in their workforce. This is extraordinary and it also means that the psychiatrists in these hospitals often need to take on the role of therapist and social worker at times, in addition to their traditional responsibilities.

As a neighboring country, the situation in Japan may provide some reference. In Japan, there were 11.87 psychiatrists, 83.81 psychiatric nurses, 3.04 psychologists, and 8.33 social workers per 100,000 population, making the shortage of mental health workers in China more pronounced (18).

The current demands for both outpatient and inpatient mental healthcare in China are growing, as measured by changes in OVs, DPs, and AHDs in these top-tier psychiatric hospitals from 2016 to 2018. Since the 1980s, meeting patients' demand in the outpatient setting in China has been a widespread problem, which is fundamentally caused by the unbalanced distribution of mental health resources and the workforce. The low-income population has increased risks of mental health problems, but little access to mental healthcare (31). In China, the 12-month rate of professional contact coverage for any mental disorders ranged from 2.7 to 3.4%, and the 12-month rate of effective treatment coverage was only 24.1% (32). Previous investigation of four provinces in China projected that 173 million Chinese adults suffer from mental disorders, of whom 158 million (91.3%) had never received professional services (5). In contrast, 18.6 million (43.1%) of such adults in the United States received professional help in 2015 (33).

China is a hierarchical society where capital cities often have the most healthcare resources. While the hospitals in the study are “large” and many trained professionals work there, the situation overall is rather bleak, especially for those in rural areas. While most well-trained professionals prefer to work and live in urban areas, the overall level of career satisfaction among mental health professionals were either moderate (general job satisfaction) or low (perceived social recognition and respect from the public and patients) and a large percentage of them considered leaving their job (20% among both psychiatrists and psychiatric nurses) (20, 34, 35).

As affiliated hospitals of medical universities or colleges, almost all the hospitals in this study were involved in psychiatric undergraduate education (90.2%, 37/41) and standardized residency training (87.8%, 36/41). In China, a qualified specialist physician is required to receive 5 years of medical (undergraduate) education and 3 years of standardized residency training (36).

A few limitations of this survey should be noted. First, the participating hospitals were all tertiary hospitals, and these hospitals often have more resources than the hospitals at the district- or county-levels, or in rural areas. According to a previous report, although provincial hospitals had 10.8% of all the psychiatric beds, they had 16.0% of mental health workers in China (50.3 and 53.6% in municipal hospitals, and 38.8 and 30.4% in county level hospitals, respectively) (15). As a result, the generalizability of our findings may be limited and our results may actually overestimate the resource allocation and workforce numbers, supporting our overall concerns. Second, we did not include some potentially important data in this survey, such as psychiatrists' subspecialty background, e.g., child psychiatrist, adult psychiatrist, or geriatric psychiatrist; and the nature of the outpatient visits (assessment, medication management, vs. therapy).

## Conclusions

In summary, we found a critical shortage of mental health professionals, particularly in less developed regions, and the situation for non-top-tier hospitals is likely worse. The staffing composition of the psychiatric workforce needs to be optimized and more psychologists and social workers must be trained and hired. More beds, especially those serving child and elderly patients are urgently needed. While improving the resources and building the workforce in psychiatric hospitals is important, developing more community-based mental health services are equally, if not more important over the long run. Psychiatric hospitals and community mental health services are part of a system that provides a continuity of care and keeps patients safe.

## Data Availability Statement

The raw data supporting the conclusions of this article will be made available by the authors, without undue reservation.

## Author Contributions

FJ, HL, YL, TL, and YLT conceived and planned the survey. LX, YZ, YS, and KZ contributed to the implementation and acquisition of data for this study. LX and FJ did all data analyses and drafted the manuscript. YLT and JR gave many useful suggestions and amended the manuscript. All authors have read and agreed to the published version of the manuscript.

## Conflict of Interest

The authors declare that the research was conducted in the absence of any commercial or financial relationships that could be construed as a potential conflict of interest.
