# Supplementary material for: Resources and Workforce in Top-Tier Psychiatric Hospitals in China: A Nationwide Survey
Source: Front Psychiatry. 2021 Feb 24;12:573333. doi: 10.3389/fpsyt.2021.573333 (PMC7943845; doi:10.3389/fpsyt.2021.573333)
Supplement: Supplementary file 1 [file Table_1.docx]

**Supplemental Table 1.** Psychiatric standardized residency training program in the 41 psychiatric hospitals

| Province^a^ | Hospital code | Teaching  Hospital (*yes or no*) | Psychiatric residency  training program  (*yes or no*) | Psychiatric residents recruited by hospitals (*n/year*)^b^ | Provincial recruitment plan  (*n/year*)^b^ |
| --- | --- | --- | --- | --- | --- |
| Liaoning | P08 | *yes* | *yes* | 16 | 104 |
|  | P34 | *no* | *yes* | 3 |  |
| Jilin | P09 | *yes* | *no* | - | 5 |
|  | P35 | *no* | *yes* | 5 |  |
| Heilongjiang | P10 | *yes* | *no* | - | 20 |
|  | P36 | *yes* | *yes* | 10 |  |
| Beijing | P01 | *yes* | *yes* | 14 | 69 |
|  | P02 | *yes* | *yes* | 25 |  |
|  | P03 | *yes* | *yes* | 30 |  |
| Tianjin | P04 | *yes* | *yes* | *N/A* | *N/A* |
| Hebei | P05 | *yes* | *no* | - | 27 |
|  | P33 | *yes* | *yes* | 5 |  |
| Shanghai | P11 | *yes* | *yes* | 42 | 51 |
| Jiangsu | P12 | *yes* | *yes* | *N/A* | *N/A* |
| Zhejiang | P13 | *yes* | *yes* | 38 | 427 |
|  | P39 | *yes* | *yes* | 0 |  |
| Fujian | P16 | *yes* | *yes* | 10 | *N/A* |
| Shandong | P18 | *yes* | *yes* | 13 | 70 |
| Guangdong | P22 | *yes* | *yes* | 47 | 100 |
| Hainan | P24 | *yes* | *yes* | 5 | 5 |
| Shanxi | P06 | *yes* | *yes* | 3 | 30 |
| Anhui | P14 | *yes* | *yes* | 5 | 57 |
|  | P15 | *yes* | *yes* | 8 |  |
| Jiangxi | P17 | *yes* | *yes* | 6 | 43 |
| Henan | P19 | *no* | *no* | - | 50 |
|  | P37 | *yes* | *yes* | 15 |  |
| Hubei | P20 | *yes* | *yes* | 15 | 69 |
|  | P40 | *yes* | *yes* | 8 |  |
| Hunan | P21 | *yes* | *yes* | 0 | 12 |
| Inner Mongolia | P07 | *yes* | *yes* | 10 | 15 |
| Guangxi | P23 | *yes* | *no* | - | 20 |
|  | P41 | *yes* | *yes* | 10 |  |
| Chongqing | P25 | *no* | *yes* | 7 | 17 |
| Sichuan | P26 | *yes* | *yes* | *N/A* | 60 |
|  | P38 | *yes* | *yes* | 8 |  |
| Guizhou | P27 | *yes* | *yes* | 6 | 15 |
| Yunnan | P28 | *yes* | *yes* | 2 | 10 |
| Shaanxi | P29 | *yes* | *yes* | 10 | 25 |
| Qinghai | P30 | *yes* | *yes* | 5 | 5 |
| Ningxia | P31 | *yes* | *yes* | 4 | 4 |
| Xinjiang | P32 | *yes* | *yes* | 6 | 12 |
| Total | - | 37/41 | 36/41 | 391 | 1322 |

^a^ The order of 29 provinces and autonomous regions: Northeast, East, Central, West China. ^b^ Data were obtained from the official websites of the platform for standardized residency training in each province or autonomous region. N/A, not available.
